# Spatial patterns and interspecific associations among trees at different stand development stages in the natural secondary forests on the Loess Plateau, China

**DOI:** 10.1002/ece3.5216

**Published:** 2019-04-30

**Authors:** Li Gu, Kevin L. O'Hara, Wei‐zhong Li, Zhi‐wen Gong

**Affiliations:** ^1^ College of Forestry Northwest A&F University Yangling China; ^2^ Department of Environmental Science, Policy & Management UC Berkeley Berkeley California; ^3^ College of Economics and Management, Research Center of Resource Economics and Environment Management Northwest A&F University Yangling China

**Keywords:** forest dynamics, interspecific association, population structure, spatial pattern

## Abstract

*Quercus wutaishansea* populations on the Loess Plateau are currently becoming more dominant in natural secondary forests, whereas *Pinus tabulaeformis* is declining. In the present paper, the diameter class (instead of age) was used to classify the different growth stages as juvenile, subadult, or adult, and the univariate function *g*(*r*) was used to analyze the dynamic changes in spatial patterns and interspecific associations in three 1‐ha tree permanent plots on the Loess Plateau, NW China. Our results suggested that the niche breadth changed with the development stage. The diameter distribution curve was consistent with the inverted “J” type, indicating that natural regeneration was common in all three plots. There was a close relationship between the spatial pattern and scale, which showed significant aggregation at small distances, and became more random as distance increased, but in the *Pinus* + *Quercus* mixed forests, the whole species were aggregated at distances up to 50 m. The degree of spatial clumping decreased from juvenile to subadult and from subadult to adult. The spatial pattern also differed at different growth stages, likely due to strong intraspecific competition. Associations among different growth stages were positively correlated at small scales. Our study is important to the understanding of the development of the *Q. wutaishansea* forests; thus, the spatial dynamic change features should be received greater attention when planning forest management and developing restoration strategies on the Loess Plateau.

## 1 INTRODUCTION

The relative spatial position of plants affects the distribution ratio of resources among individuals, which is one of the key factors determining the intraspecific and interspecific competition (Harms, Condit, Hubbell, & Foster, 2001). Spatial patterns among tree species within forest stands are a result of long‐term interactions between the tree community and environment, which could affect species coexistence and community structure. The spatial pattern of plant populations has spatial autocorrelation, and the variation rule is closely related to spatial scale (Yang, Vázquez, Feng, Liu, & Zhao, 2018). Changes in spatial patterns often begin with the aggregation of young individuals that develop into random or evenly uniform patterns as they developed (Getzin et al., 2006). Spatial aggregation may be a consequence of many diverse factors, which include regeneration strategies such as seed dispersal, clonal propagation, soil conditions, and disturbances that affect the canopy structure (Larson et al., 2016; Noma, 1997; Yousef & Krzysztof, 2017). In contrast, regular spatial patterns are often attributed to local density‐dependent influences, which include the mortality caused by species‐specific natural enemies and competition from conspecific neighbors (Fibich, Leps, Novotny, Klimeš, & Těšitel, 2016; Tanner, Hughes, & Connell, 1994). The spatial patterns of trees may also be influenced by biotic and abiotic factors during stand development, and the appropriate disturbance‐based silvicultural strategies could be designed to influence forest dynamic processes (Whitfeld et al., 2014).

Interspecific associations could indicate the spatial distribution relationship and functional dependency of species and their environment (Su et al., 2015), which is central to successional theory. Complex and stable forest communities result from the replacement and establishment of different tree species during stand development, and changes in tree size may be informative in the study of developmental changes across forests because of competition among similarly sized individuals (Moeur, 1993; Salas, Lemay, Nunez, Pacheco, & Espinosa, 2006). Several models have implicitly assumed that interspecific associations are of primary importance in determining stand development patterns (Janík et al., 2016; Nakashizuka, 2001). The arrangement of tree species could provide significant information about forest dynamics processes and inter‐ and intraspecific interactions (Alekseev & Zherebtsov, 1995), including forest establishment, tree growth, species competition, plant reproduction, and mortality (Martíneza, Wieganda, Taboadab, Fernando, & Joséramón, 2010; Schleicher, Wiegand, & Ward, 2011). Understanding the interactions can provide important insights for both forest dynamics and management (Alekseev & Zherebtsov, 1995; O'Hara, 2014; Oliver & Larson, 1996).

**Figure 1:**
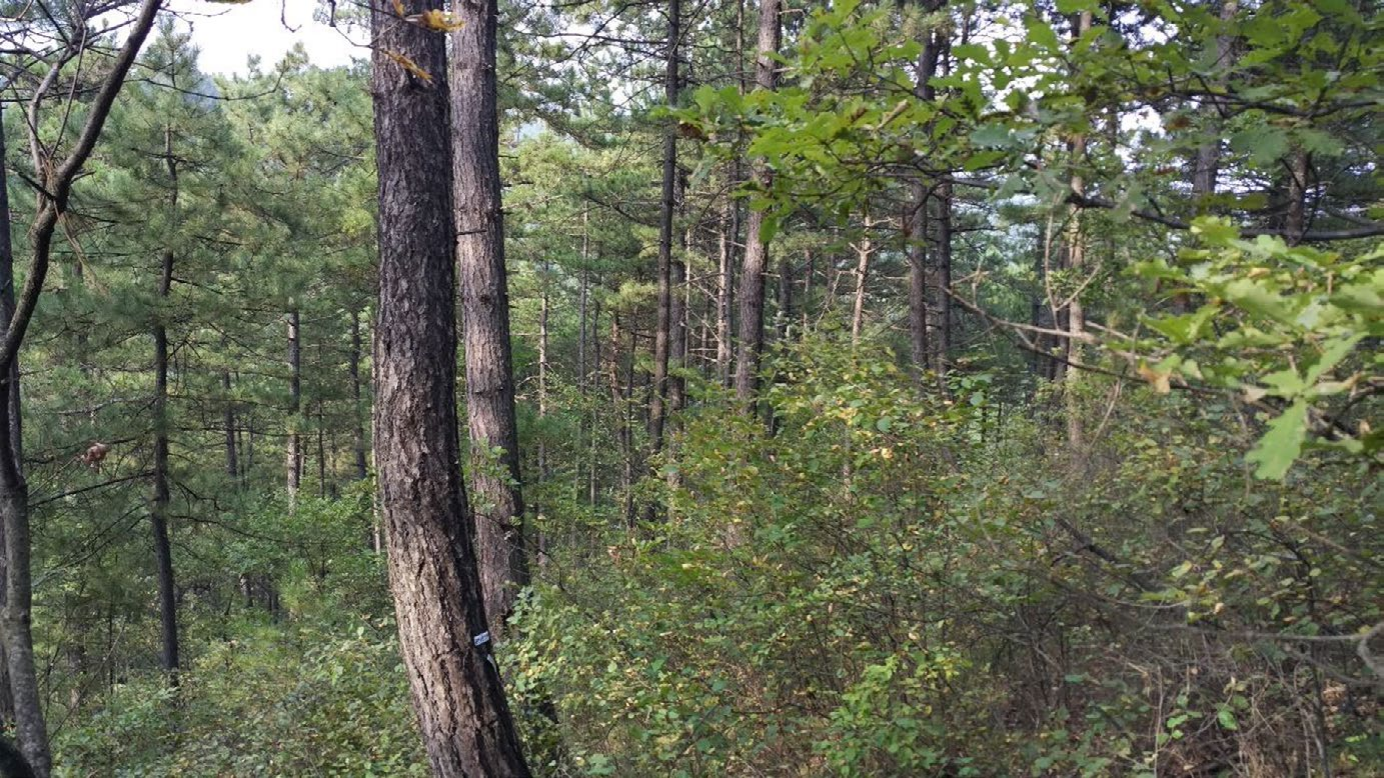
An organism photograph: this figure is for *Quercus wutaishansea*forests, *which is* the dominant or natural climax forest in the arid and semiarid soils of the Loess Plateau, China

The Loess Plateau is located in the northwestern China and is one of China's four large plateaus. This plateau is one of the birthplaces of ancient civilizations in China and features the most concentrated distribution and largest areas of loess on Earth. Most importantly, the Loess Plateau contains some of the largest areas of soil erosion and possesses one of the most vulnerable ecosystems in the world (Sun et al., 2015). Reforestation or natural forest regeneration can be an effective measure in controlling the soil erosion and thus in ameliorating the ecological environment. During reforestation or natural forest regeneration, internal competition among plants for limited resources has been proven to be the driving factor for forest succession. *Quercus wutaishansea* forests are the dominant or natural climax forest in the arid and semiarid soils of the Loess Plateau, China (Figure 1; Cheng, Han, & Kang, 2014), and the *Q. wutaishansea* species have cold tolerance and drought tolerance (Gao & Sun, 2005). Studies of vegetation variation and development have shown that, after herb and shrub communities, *Pinus tabulaeformis,* as a pioneer species, is generally the first established species and captures resources in disturbance openings (Fan, Guo, Wang, & Duan, 2014). The high recruitment rates of *P. tabulaeformis* are due to the high light intensity and temperatures of the bare land, and the seedlings are competitive, especially on the burnt ground (Gomez, 2004; Yang et al., 2018). Once *P. tabulaeformis* forests are formed, other, relatively shade‐tolerant, species (i.e., *Q. wutaishansea*) are able to invade (Collins & Carson, 2004). The two species were found in a positive self‐correlation and high intensity of competition (Li et al., 2008), which is consistent with those by Tilman (1994) and Pedersen et al. (2012) who found that the competition intensity generally decreased with the growth of trees.

The opportunities for *Q. wutaishansea* to establish under the *P. tabulaeformis* were documented on the Loess Plateau, where a key strategy was establishment of shade‐tolerant advanced reproduction (Rentch, Fajvan, & Rrjr, 2003). In the past 50 years, the *P. tabulaeformis* forest on the shady and semishady slope has been or is developing the *Q. wutaishansea* forest on the Loess Plateau. It has been suggested that pure *P. tabulaeformis* and *Q. wutaishansea* forests are at different stand development stages (Dang, Wang, & Wang, 2002), and the natural transformation of *P. tabulaeformis* forests to mixed *P. tabulaeformis* and *Q. wutaishansea* forests has significant impacts on the underlying ecological interactions of the main species. Thus, a detailed investigation of forest spatial distributions may demonstrate and quantify the population mechanisms in natural secondary forests on the Loess Plateau. In this paper, we compare the composition, structure, spatial patterns, and interaction of dominant tree species among three different forest communities on the Loess Plateau. The objectives of this study were to determine how spatial patterns or aggregation change with scale and size class through the development process, and gain insight into tree coexistence and development trends. These results will provide the basis for future ecological restoration research and forest reconstruction, and offer a valuable reference for future planning of afforestation and ecological policies on the Loess Plateau and similar areas.

## 2 MATERIALS AND METHODS

### 2.1 Study area

**Figure 2:**
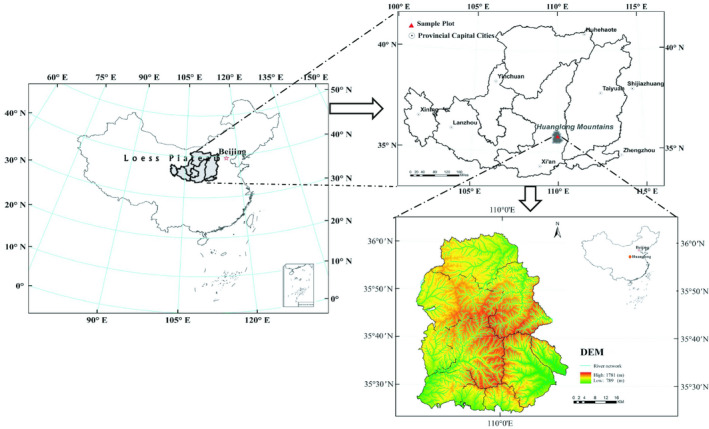
The location of the study area

Our study area was located on the southeastern Loess Plateau in northern Shaanxi Province. Detailed surveys were conducted on the Huanglong Mountains (Figure 2). The entire forest zone shows extreme irregularity, a complicated geological structure, and fractured landforms, with the typical geographical structures of the Loess Plateau. The altitude ranges from 800 to 2,500 m, the frost‐free period averages 175 days, annual precipitation is 611.8 mm (mostly from July to September), and the general climate is within the warm temperate zone (Guo, Li, He, Yang, & Sun, 2011). The steep and hilly topography mainly consists of granite and gneiss. The mean slope is 35°, and the mean soil depth is 50 cm. The soil is classified as mountain brown earth (Shi et al., 2004). The plants on the Loess Plateau consist of North China flora, with more than 580 plant species and 46 tree species in 29 genera and 22 families. The main tree species include *P. tabulaeformis*, *Betula platyphylla*, *Populus davidiana*, *Q. wutaishansea*, *Acer ginnala*, *Pyrus betulaefolia*, *Toxicodendron vernicifluum*, *Populus simonii*, *Prunus davidiana*, *Juglans cathayensis*, and *Betula albosinensis*.

### 2.2 Data sets

Substituting space for time has been a widely used method for evaluating vegetation changes. It was initially used to distinguish successional stages in woody vegetation, and the method assumed that time was the only difference (Pickett, 1989). According to the space‐time method, in August 2013 and August 2014, three permanent sample plots 1 ha (100 × 100 m) were established in the natural secondary forest on Huanglong Mountains. Three plots with typical characteristics in the late stage of the natural secondary changed series were arranged, that is, Plot I for *P. tabulaeformis* forests; Plot II for *Pinus* + *Quercus* mixed forests; and Plot III for *Q. wutaishansea* forests. Each forest was sampled on both a southern slope and a northern slope (with a due east‐due west line as the boundary, the slope at the southern boundary is the sunny slope and the slope at the northern boundary is the shaded slope). With a canopy density of approximately 0.8 based on the adjacent gridding method, the plot was divided into 25 20 × 20 m small plots. In each small plot, the coordinates (*x* and *y*), diameter at breast height in 1.3 m (DBH), height (*h*), and the crown width of adult trees (DBH >3 cm) were measured (Table 1). In addition, 5 2 × 2 m^2^ bush quadrats and 5 1 × 1 m^2^ plant quadrats (1 × 1 m) were, respectively, arranged at the four corners and at the center of the subplot.

**Table 1:** The basic information of the samples

Plot number	Coordinates	Area (ha)	Elevation (m)	Slope (°)	Average DBH (cm)	Average basal area (m^2^/ha)	Tree number (n/ha)	Age	Canopy cover
Plot I	109°46′, 35° 40′	1	1,351	31°	16.9	20.3217	911	52	0.8
Plot II	109°58′, 35° 37′	1	1,430	26°	12.1	23.5662	2,051	47	0.8
Plot III	109°57′, 35° 31′	1	1,505	29°	15.7	19.1445	985	53	0.8

Note

Plot I for *Pinus tabulaeformis* forests; Plot II for *Pinus + Quercus* mixed forests; and Plot III for *Quercus wutaishansea* forests.

We classified all individual trees into three size classes based on our sample survey, combined with a static population life table: “juvenile” (3 ≤ DBH < 10), “subadult” (10 ≤ DBH < 20), and “adult” (DBH ≥ 20) (Wang, Li, & Cao, 2016). Saplings <1.3 m tall were included.

### 2.3 Data analysis

Niche breadth is considered to be the determinants of species diversity and community structure, which was calculated using the Shannon index (Leibold, 1995; Magurran, 1988):

$$B_{i}=-\sum_{j=1}^{r}p_{\mathrm{ij}}*\mathrm{lg}p_{\mathrm{ij}}$$

where *B_i_* is the niche breadth of the *i*th species, with the range as [1/*r*, 1], *r* is the total number of resource states, and *p_ij_* is the proportion of the individuals of the *i*th species, which is associated with resource state *j*.

The importance value is a comprehensive quantitative indicator used to characterize the status and role of each species in the community, the larger the importance value of a species, the greater the dominance of the species in the plot, which was calculated with the following equation (Li et al., 2006):

$$\text{IV}=\frac{\text{RD}+\text{RF}+\text{RA}}{3}$$

$$\text{IV}=\frac{\frac{n_{i}}{\sum_{i=1}^{S}n_{i}}*100+\frac{a_{i}}{\sum_{i=1}^{S}a_{i}}*100+\frac{f_{i}}{\sum_{i=1}^{S}f_{i}}*100}{3}$$

where RD is the relative dominance, RF is the relative dominance, RA is the relative dominance, *n_i_* is the number of individuals of the ith species, *a_i_* is the basal area at the height of 1.3 m belonging to the *i*th species, *f_i_* is the number of quadrats in which the *i*th species appeared, and *S* is the total number of species.

Ripley's K function *K*(*r*) is an important spatial pattern statistic, which is a cumulative distribution function within the distance of *r* (Getis & Franklin, 1987; Greig‐Smith, 1952; Ripley, 1977). An alternative approach uses rings (or annulus) instead of circles, with a pair correlation function *g*(*r*) or the O‐ring statistic *O*(*r*) (Wiegand, Moloney, Naves, & Knauer, 1999). The advantage of this alternative approach is that one can isolate specific distance classes, whereas the *K*(*r*) confounds effects at larger distances with effects at shorter distances (Condit et al., 2000; Stoyan & Penttinen, 2000).

In the present study, the univariate function *g*(*r*) was used to analyze the spatial distribution patterns in the categories of all trees and four size classes of trees. The function *g*(*r*) is related to Ripley's K function (Wiegand, Jeltsch, & Ward, 2000):

$$K\left(r\right)=n^{2}\left|A\right|\sum_{i\neq j}\sum e_{\mathrm{ij}}^{-1}\left(u_{\mathrm{ij}}\right)$$

$$g\left(r\right)=\frac{K^{\prime }\left(r\right)}{2\pi r}$$

where *r* is the distance (ring in *g*(*r*)) rad, *A* is the area of study plot, *u_ij_* is the distance between the focal tree (*i*) and its neighboring tree (*j*), *n* is the total number of points in the point pattern, *u_ij_* = 1, if *u_ij_* < *r* and 0 otherwise, and *e_ij_* is the weighting factor for eliminating edge effect correction.

For the univariate function *g*(*r*), the spatial scale was 0–50 m, and we used a ring width of one meter and used 199 Monte Carlo simulations of complete spatial randomness (CSR) to acquire pointwise critical envelopes, and the significance level was 0.01 (namely *p* ≤ 0.01). If the value of *g*(*r*) was above (or below) the upper limit of the confidence envelope, the spatial pattern indicates the clustered (or regular) pattern at a given distance *r*, and within the confidence intervals indicated random pattern.

To investigate the relationships between different life stages, we used bivariate pair correlation function *g*_12_(*r*), which is the extended *g*(*r*) function to multitype point patterns. *g*_12_(*r*) can be defined as the expected number of trees of life stage 2 at spatial scale *r* of an arbitrary tree of life stage 1, divided by the intensity of life stage 2 (Stoyan & Penttinen, 2000).

$$g_{12}\left(r\right)=\frac{1}{2\pi r}\frac{A}{n_{1}n_{2}}\sum_{i=1}^{n_{1}}\sum_{j=1}^{n_{2}}u_{\mathrm{ij}}\left(e_{\mathrm{ij}}\right)$$

where *u_ij_* is the distance between the focal tree of pattern 1, and its neighboring tree of pattern 2, *n*_1_ and *n*_2_ are the total numbers of trees in the patterns 1 and 2, respectively (Wiegand & Molone, 2004).

For *g*_12_(*r*), we also used a ring width of one cell and used 199 Monte Carlo simulations of complete spatial randomness (CSR) to acquire pointwise critical envelopes and the significance level was 0.01 (namely *p* ≤ 0.01). The spatial associations between different growth stages were examined with the independent null model. If the value of *g*12(*r*) was above the upper (or below the lower) confidence limit, the relationship indicates that species are positively (or negatively) associated at the distance *r*, and within the confidence intervals indicated no interaction (Thioulouse, Chessel, Doledec, & Olivier, 1997; Wiegand et al., 2000).

We used package “spatstat” in R‐3.4.2 to conduct all spatial analyses. In our analyses, a 1 m cell size was used, which preliminary analysis had suggested was a sufficient resolution to address our questions.

## 3 RESULTS

### 3.1 Forest composition and structure

We identified 23 species, belonging to 12 families among the 911 live trees found in the 25 quadrats in *P. tabulaeformis* forests on the Loess Plateau. Twenty species, belonging to 14 families, respectively, among the 2,051 and 985 live trees were found in the 25 quadrats in* Pinus* + *Quercus* mixed forests and *Q. wutaishansea* forests (Tables 1 and 2). Twelve species were ubiquitous across all forests, and three of these were dominant species with importance values >5 (Table 2): *P. tabulaeformis*, *Q. wutaishansea*, and *Po. davidiana*.

**Table 2:** Ecological characteristic of dominant tree species (IV > 5) in the three 1‐ha plots

Species	Niche breadth	Relative dominance	Relative frequency	Relative abundance	Importance value
Plot Ⅰ					
*Pinus tabulaeformis*	1.36	0.86	0.22	0.61	56.14
*Quercus wutaishansea*	1.30	0.07	0.20	0.22	16.17
*Betula platyphylla*	1.19	0.03	0.17	0.08	9.41
*Populus davidiana*	1.11	0.02	0.13	0.03	6.05
*Swida macrophylla*	0.97	0.01	0.11	0.03	5.04
Plot II					
*P. tabulaeformis*	1.33	0.33	0.16	0.39	29.50
*Q. wutaishansea*	1.33	0.29	0.16	0.26	24.00
*Lacquer tree*	1.32	0.30	0.16	0.24	23.27
*Pyrus betulaefolia*	1.24	0.02	0.14	0.02	6.13
*Po. davidiana*	1.12	0.03	0.11	0.03	5.80
*B. platyphylla*	1.36	0.02	0.12	0.03	5.61
Plot III					
*Q. wutaishansea*	1.36	0.87	0.19	0.68	57.99
*P. tabulaeformis*	1.21	0.03	0.17	0.10	10.06
*Acer mono*	1.02	0.03	0.11	0.07	6.81
*Swida macrophylla*	1.02	0.02	0.13	0.03	6.01
*Po. davidiana*	1.14	0.00	0.11	0.06	5.67

Note

IV is the Importance value, Plot I for *P. tabulaeformis* forests; Plot II for *Pinus + Quercus* mixed forests; and Plot III for *Q. wutaishansea* forests.

The niche breadth changed regularly with stand development. Before the invasion of *Q. wutaishansea*, *P. tabulaeformis* had the greatest niche breadth (1.36). The importance value of *P. tabulaeformis* was highest in *P. tabulaeformis* forests (56.14) and lowest in *Q. wutaishansea* forests (10.06; Table 2). The importance value of* Q. wutaishansea* varied noticeably from 16.17 in *P. tabulaeformis* forests to 57.99 in *Q. wutaishansea* forests (Table 2). These total importance values of two species were higher than those of any other species across all three forests, indicating that they were the dominant species.

**Figure 3:**
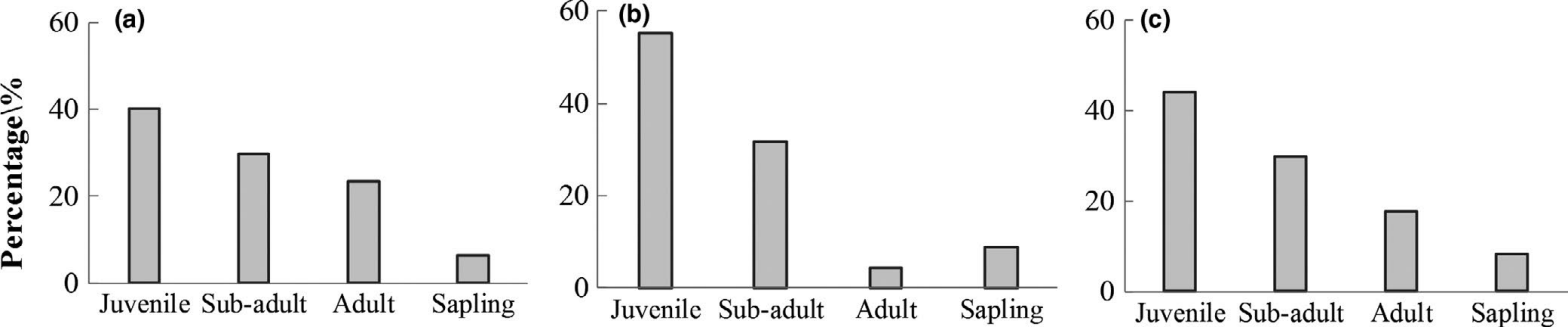
Dbh class distributions of all trees in each 1‐ha plot. In this Figure, *x*‐axes were displayed the four size classes, and the *y*‐axes were displayed the percentage proportion of individual trees. (a) *Pinus tabulaeformis* forest, (b) *Pinus + Quercus* mixed forests, and (c) *Quercus wutaishansea* forest. The four size classes were “Juvenile” (3 < DBH < 10), “Subadult” (10 < DBH < 20), “Adult” (DBH ≥ 20), and “Sapling” is with heights <1.3 m was included

**Figure 4:**
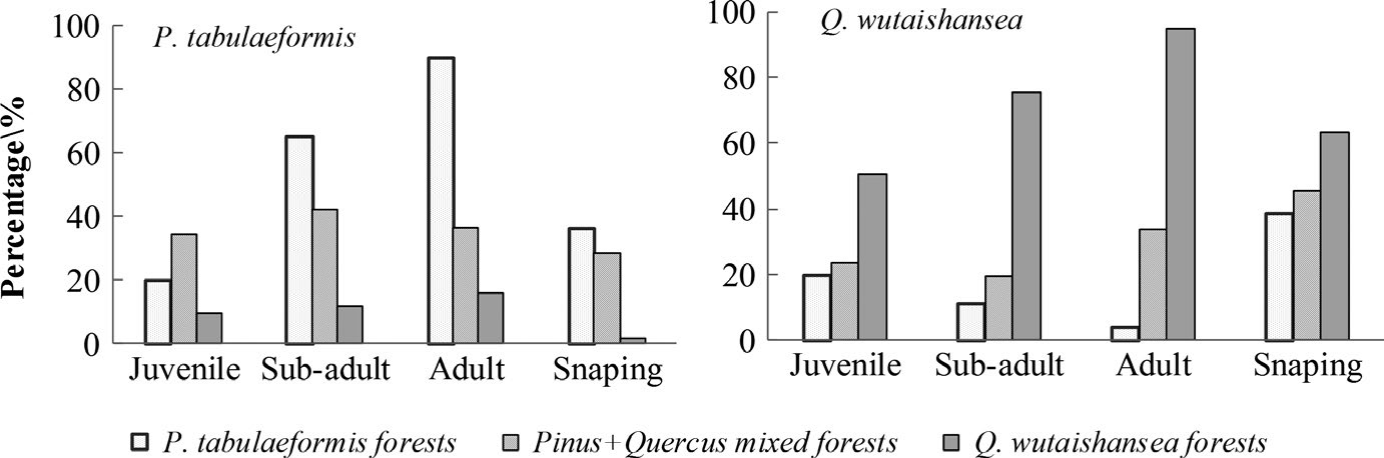
Dbh class distributions of *Pinus tabulaeformis* and *Quercus wutaishansea* in each 1‐ha plot. In this Figure, *x*‐axes were displayed the four size classes, and the *y*‐axes were displayed the percentage proportion of individual trees. The four size classes were “Juvenile” (3 < DBH < 10), “Subadult” (10 < DBH < 20), “Adult” (DBH ≥ 20), and “Sapling” is with heights <1.3 m was included

The diameter class distribution showed that the numbers of individuals were gradually decreasing as the diameters increased (Figure 3). The diameter structure was relatively unitary and indicated that juvenile trees, ranging in diameter from 3.0 to 10.9 cm, accounted for trees in each stand. In *P. tabulaeformis* forest, the quantities of saplings for *P. tabulaeformis* and *Q. wutaishansea* were more than juveniles; and in *Pinus* + *Quercus* mixed forests and *Q. wutaishansea* forests, and sapling for *Q. wutaishansea* was more than juveniles (Figures 3 and 4). Dbh class distributions of *P. tabulaeformis* and *Q. wutaishansea* in each 1‐ha plot indicated the natural regeneration ability of *Q. wutaishansea* was stronger than *P. tabulaeformis*, which was also related to the negative tolerance of *Q. wutaishansea* saplings.

### 3.2 Spatial patterns at development stages

**Figure 5:**
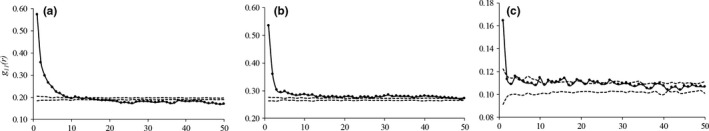
Spatial patterns of all trees in the different successional stages using point pattern analysis method *g*(*r*): (a) *Pinus tabulaeformis* forest, (b) *Pinus + Quercus* mixed forests, (c) *Quercus wutaishansea* forest. We used 199 Monte Carlo simulations of complete spatial randomness (CSR) to obtain pointwise critical envelopes for *g*(*r*). We considered the pointwise Monte Carlo test significant at *p* ≤ 0.01. If *g*(*r*) within confidence intervals, then the spatial pattern at distance *r* is entirely random; if *g*(*r*) is above the upper confidence interval and below the lower confidence interval, then the spatial pattern indicates aggregated pattern and regular pattern, respectively (*p* < 0.01)

The spatial patterns among different development stages were estimated by the pair correlation function (Figure 5). In the forest dominated by *P. tabulaeformis*, species distribution patterns transitioned from aggregated to random to uniform. Additionally, forests displayed an intensive aggregation pattern of small‐scale at distances up to 14 m, a random pattern at distances from 15 to 19 m, and a uniform pattern above 19m. In *Pinus* + *Quercus* mixed forests, all trees were markedly aggregated at all distances (*p* < 0.01). In *Q. wutaishansea* forests, trees were aggregated at distances from 0 to 28 m and tended to display a random pattern at greater distances.

### 3.3 Spatial patterns among life stages

**Figure 6:**
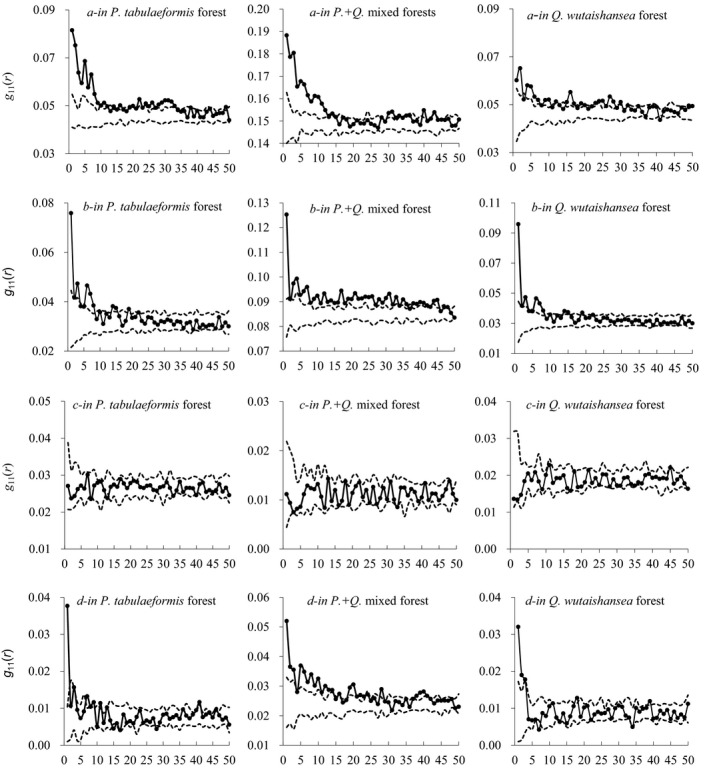
Spatial patterns of the four size classes analyzed at the different growth stages, which were Juvenile stage (a), Subadult stage (b), Adult stage (c), and Sapling stage (d)

We next analyzed the spatial distributions in different tree size classes (Figure 6). In all three forests, juvenile trees showed an aggregated pattern at distances up to 10 m. In *P. tabulaeformis* forests and in *Q. wutaishansea* forests, juveniles displayed a random pattern at greater distances. In *Pinus + Quercus* mixed forests, however, juveniles showed an aggregated pattern up to distances of 13 m, and random between 29 and 36 m, and the value of *g*(*r*) was close to the upper broken fitting line.

The pattern of subadult trees in *P. tabulaeformis* forests and in *Q. wutaishansea* forests were aggregated at distances of 1–8 m; and at distances >8 m, distribution patterns were random. In *Pinus* + *Quercus* mixed forests, however, subadults were randomly distributed at all distances (*p* < 0.01).

Distribution of adult trees was random in all three forests over all distances. Saplings were randomly distributed in *P. tabulaeformis* forest and in *Q. wutaishansea* forests, but in *Pinus* + *Quercus* mixed forests displayed an aggregated pattern that declined with increasing distance.

### 3.4 Spatial associations

**Figure 7:**
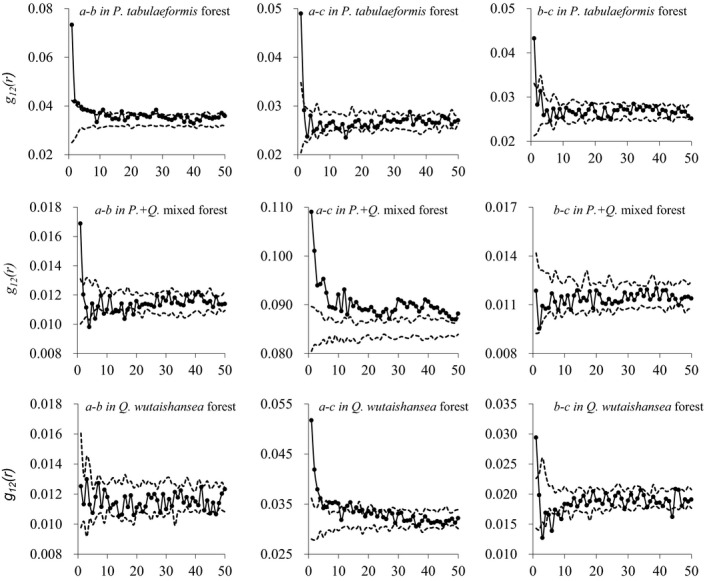
The bivariate statistic of the pair correlation function was used to analyze the spatial associations of the four size classes analyzed at the different growth stages at scales 0–50 m, which were Juvenile stage (a), Subadult stage (b), and Adult stage (c)

Using bivariate statistics, we performed species associations among life stages in all forests at distances from 0 to 50 m. In *P. tabulaeformis* forests, our results suggested a positive association between juveniles and subadults from distances of 0–12 m, and no spatial associations between 23 and 50m (Figure 7). Similarly, adults were positively spatially associated with juveniles and subadults at distances <1 m, but at other distances they were spatially independent. In *Pinus + Quercus* mixed forests, juveniles were negatively associated at low distances and random at high distances. Regardless of distance, spatial relationship showed all random patterns between adults and subadults. Juveniles and adults had a positive association across all distances. In *Q. wutaishansea* forests, juveniles showed random pattern with subadults at all distances. We observed few significant relationships between juveniles and adults. Subadults were spatially independent at distances <2 m and at the distances between 10 and 50 m. Between 3 and 6 m, subadults were negatively associated with adults.

## 4 DISCUSSION

### 4.1 Changes in population structure

It has been demonstrated that tree size was an important factor that affected stem growth and could be used to explain forest dynamics (Jang, Christopher, & Lim, 2013; Zhang, Gove, Liu, & Leak, 2001). In our research, the tree diameter class distribution curves across all three forests suggested that trees in all forests were highly dense as saplings, but that mortality was also high (Figure 3). It has been shown that initial clustering structure for juveniles was an important factor affecting adult spatial structure (Zhao, Wang, Bai, Pan, & Wang, 2015), but environmental heterogeneity also was an important factor (Myster & Pickett, 1992; Schurr, Bossdorf, Milton, & Schumacher, 2004). In *Pinus* + *Quercus* mixed forests, tree density was 2,051 n/ha, more than twice the density of the other two forests. This indicated that competition was more intense in *Pinus* + *Quercus* mixed forests, affecting stand growth and tree distribution.

Changes in environmental adaptability during population development determined the trend and rate of community development. The study on the niche breadth at different forest development stages showed that the niche breadth of *Q. wutaishansea* and *P. tabulaeformis* were different in the same habitat. In *Q. wutaishansea* forests, the niche breadth of *Q. wutaishansea* was larger and more strong adaptability, and there was no adult *P. tabulaeformis*, indicating that the tree species of *P. tabulaeformis* cannot replace *Q. wutaishansea* forest; in *Pinus* + *Quercus* mixed forests, the niche breadth of *P. tabulaeformis* was not much different from that of *Q. wutaishansea*, indicating that both *P. tabulaeformis* and *Q. wutaishansea* can adapt well to the environment, and *P. tabulaeformis* forest would be stable for a long time. But with further development, it would eventually form a mixed forest dominated by *Q. wutaishansea*. This was consistent with the results of the change and development of vegetation in the past 50 years in the same area by Zou, Liu, and Wang (2002), Zou, Cheng, and Lei (1998) and Lei, Wang, Guo, and Zhu (2007).

It has been demonstrated that *Q. wutaishansea* is more shade tolerant than *P. tabulaeformis* (Zhu, 1999). This enabled *Q. wutaishansea* to persist in the shade of *P. tablulaeformis*, thereby forming more complex structures than when these species were growth separately. The population structure changed markedly between *P. tabulaeformis* forests and *Q. wutaishansea* forests, as expected. The population of *Q. wutaishansea* in this unmanaged secondary natural forest was characterized by increased numbers of individual stems, recruitment, and population growth of *Q. wutaishansea*, and *Q. wutaishansea* was the dominant tree species in these forests, while the numbers of specimens of *P. tabulaeformis*, traditionally important in secondary natural forest, sharply decreased. *Quercus wutaishansea* had a tendency to dominate these natural secondary forests, while *P. tabulaeformis* persisted only on sunny sites. The seedlings of *Q. wutaishansea,* with their shade tolerance, were more competitive for growing under the canopy (Fan et al., 2014). The net effect of these processes was a long‐term reduction in *P. tabulaeformis* with consequences for the composition and structure of these forests (Gomez, 2004; Zou et al., 1998). There was a large‐scale artificial *P. tabulaeformis* forest in the study area, and reasonable human intervention will gradually develop into the *Pinus + Quercus* mixed forests, and then develop into *Q. wutaishansea* forests (Wang, 1991).

### 4.2 Spatial distribution

Our comparisons of spatial structure among different development stages suggested that stages differed greatly in spatial pattern. Spatial patterns of pioneer species had less aggregation in older forests or in the less disturbed forests, as compared to forests that were younger or more disturbed. The action of competition had been shown to decrease aggregation and promoted shifting toward more regular or random patterns (López, Alcázar, & Ruiz, 2010; Martíneza et al., 2010).

Population distribution patterns depended significantly on spatial scale as has been reported elsewhere (Levin, 1992). We found that *P. tabulaeformis* forests exhibited small‐scale aggregation and tended to form random patterns at large scales. This may be due to the lack of airbags, wings, and other flight aids; it was difficult to spread the seeds over long distances with the help of wind, and the seeds were concentrated around the mother plants. The complex terrain in the mountains also concentrates seeds in low places (Yang et al., 2018). This result was the same as previous studies, particularly other coniferous species which were aggregated in small scales and a randomly distribution in large scales (Cain & Shelton, 1995; Koukoulas & Blackburn, 2005; Shibata, Kikuchi, Tanaka, Sueyoshi, & Yoshimaru, 2009).

Under natural conditions, different tree sizes were typically found in separate canopy strata. Since stands may be subject to variable environmental conditions, this may affect distribution patterns (He, Legendre, & LaFrankie, 1997). We found that different stages of development had many juveniles which showed aggregated distributions at small scales. Aggregated spatial distributions are usually observed in naturally regenerating forests (Wang, Shao, & Shangguan, 2010), particularly in arid and semiarid forests (Xu, Xu, & Xie, 2012). We found evidence of clumping in *Pinus* + *Quercus* mixed forests. However, the degree of aggregation varied in forest types, and the distribution of aggregation could be explained by regeneration processes. Regeneration typically occurred near seed sources and was influenced by habitat heterogeneity (Jang et al., 2013).

At larger scales, the spatial patterns in these forests lost their strong aggregation and approached random for both juveniles and subadults. Unlike juveniles and subadults, random patterns were observed for adult trees at almost every scale, which may be because of intraspecific thinning due to high resource requirements and competition (Halpin & Lorimer, 2016; Tiphaine, Hugo, Frédérik, & Yves, 2014). Species that show aggregated patterns as young trees tend to present random patterns as the forest becomes more stable (Aldrich, Parker, Ward, & Michler, 2003). Our results indicated that density‐dependent mortality of offspring was common. When dense plant communities are subject to intense competition, density‐dependent mortality occurs in the process known as self‐thinning (Yoda, Kira, Ogawa, & Hozumi, 1963). However, self‐thinning, especially in natural forests, does not necessarily lead to random spatial patterns. For example, catastrophic natural events such as strong winds may result in random spatial patterns of adult *P. tabulaeformis*, which were more sensitive to wind damage due to shallow, wide root systems (Gao & Sun, 2005). However, such catastrophic events were rare and produce only local damage, so it was not easy to ensure that they would affect spatial patterns at a broad scale.

It was notable that the most limiting condition in the areas of semiarid and arid was water. Changes in forest cover (Whitford, 2003), soil surface cover, and texture affected water available to trees (Zou, Li, Xu, & Xu, 2010). For natural secondary forest, small‐scale spatial patterns were mainly determined by biological characteristics such as species origin, initiation, and seed dissemination. In contrast, as spatial scale increased, the influence of topography, geomorphology, light, and moisture gradually replaced biological characteristics in importance, resulting in an alteration in spatial distribution patterns. These results lay the foundation for understanding the coexistence mechanism of major species in natural secondary forests and the impact of artificial replanting of forest stands. In the case of near‐natural afforestation and management, it is necessary to pay attention to maintaining reasonable planting and growth density within and between species.

### 4.3 Associations among life stage

Associations across life stages were mostly positive, and small‐scale, probably attributed to prompt growth reactions to spatially limited light availability, which was the same opinions suggested by Hubbell, Ahumada, and Condit (2001). However, the positive association that we observed among adult trees and juvenile trees in *P. tabulaeformis* forests were somewhat surprising, as this pattern would be expected to vanish due to competition as trees grew to adult stage. Our results were in accordance with the findings of recent investigations (Martíneza et al., 2010). Additionally, Pacala (1997) suggested that intraspecific competition effects should be much more important than interspecific competition, which could promote species coexistence (Carson, & Pickett, 1990; Martíneza et al., 2010; Zhao, Kang, Guo, Yang, & Xu, 2012). It was interesting that the forests demonstrated self‐thinning processes of intraspecific species, which could create favorable conditions for the invasion and settlement of other species. In order to promote the nature regeneration, the shrubs under the forest can be properly cleaned to provide more space for the sapling growth. At the same time, it is also possible to selectively harvest poorly growing trees and diseased trees to promote forest renewal. Moderate intervention on population distribution and adopting tending measures could promote forest succession.

Our analyses were based only on comparisons of spatial patterns and interspecific associations. However, it was noted that the 1‐ha sample plots were representative of typical secondary natural forest composition, as compared to replicated forest series at smaller scales (Feroz, Yoshimura, & Hagihara, 2008; Whitfeld et al., 2014). As there were similar factors in environmental heterogeneity among the three forests, the spatial pattern differences were likely caused by other factors. In *P. tabulaeformis* forests, competition among neighboring individuals influenced spatial patterns to a greater extent and then in the *Pinus* + *Quercus* mixed forests for a long time.

Our findings were somewhat expected, as interspecific differences in shade tolerance among tree species are key determinants of forest dynamics and structure, and the shade under the canopy of shade intolerant species is expected to facilitate establishment and growth of shade‐tolerant species (Oliver & Larson, 1996). Therefore, young individuals of *Q. wutaishansea* would gradually dominate the natural space after a period of competition and elimination. Artificial disturbances, such as thinning or tending might accelerate this process, improve internal structure of forest and be conducive to forest development. Many factors influence the formation of forests, and no spatial pattern analysis can capture all of these factors. Therefore, to gain a better understanding of spatial pattern formation at different stages and to provide a framework for local forest management, the relationship among species (including *P. tabulaeformis* and *B. platyphylla*) and environmental factors should be further examined.

## 5 CONCLUSIONS

Comparisons of spatial structure among our three forest types suggested that the most important influences on spatial patterns were the different forest development stages. Our study found that the differences in spatial patterns among various stages could inform mechanistic hypotheses explaining successional development. The seedlings of *P. tabulaeformis* were light ‐demanding and grew quickly with increasing light availability, while the seedlings of *Q. wutaishansea* were shade tolerant. Knowledge of this pattern can help to improve silvicultural practices. But due to the limited experimental conditions, the data in this study did not include trees with a DBH <3.0 cm. Therefore, discrepancies with other studies in the spatial distribution patterns and associations may be observed.

Stand development was difficult to evaluate because of its long timescale. Nevertheless, the length and structure of each stage in this study contributed to the site conditions, vegetation, and disturbance characteristics. Regenerated seedlings of *Q. wutaishansea* were found on slopes in *P. tabulaeformis* forests, and the proportion was more than that of *P. tabulaeformis*. In some stands, *Q. wutaishansea* occupied the second sublayer of the tree layer, demonstrating a notable tendency to replace *P. tabulaeformis*. In the process of forest management, artificial measures such as updating and arranging tree species can be adopted to accelerate the development of forests. It is worth noting that the mechanism of community stability structure needs to be further studied. The self‐renewal of dominant species, the coexistence of species, and the community succession status of tree species needs to be long‐term oriented observations.

## CONFLICT OF INTEREST

None declared.

## AUTHOR CONTRIBUTION

Li Gu prepared figures and tables and wrote the main manuscript text; Kevin L. O'Hara was mainly responsible for the content of the thesis, revision, and language adaptation; Weizhong Li was responsible for field survey and data collection; Zhiwen Gong was the corresponding author. And all authors reviewed the manuscript.

## Data Availability

Data available from the Dryad Digital Repository: https://doi.org/10.5061/dryad.hk31vm7. Please see lines 397.

## References

[ece35216-bib-0001] Aldrich, P. R. , Parker, G. R. , Ward, J. S. , & Michler, C. H. (2003). Spatial dispersion of trees in an old‐growth temperate hardwood forest over 60 years of succession. Forest Ecology and Management, 180(1–3), 475–491. 10.1016/S0378-1127(02)00612-6

[ece35216-bib-0002] Alekseev, A. S. , & Zherebtsov, R. R. (1995). Regularities of spatial distribution of damaged vegetation under conditions of regional and local air pollution (with reference to the impact zone around the Pechenganikel mining and smelting plant). Russian Journal of Ecology, 26(6), 428–435.

[ece35216-bib-0003] Carson, W. P. , & Pickett, S. T. A. (1990). Role of resources and disturbance in the organization of an old‐field plant community. Ecology, 71(1), 226–238. 10.2307/1940262

[ece35216-bib-0004] Cain, M. D. , & Shelton, M. G. (1995). Thirty eight years of autogenic, woody understories dynamics in a mature, temperate pine‐oak forest. Canadian Journal of Forest Research, 25(12), 1997–2009. 10.1139/x95-216

[ece35216-bib-0005] Cheng, X. Q. , Han, H. R. , & Kang, F. F. (2014). Point pattern analysis of different life stages of Quercus liaotungensis in Lingkong Mountain, Shanxi Province, China. Journal of Plant Interactions, 9(1), 233–240.

[ece35216-bib-0006] Collins, R. J. , & Carson, W. P. (2004). The effects of environment and life stage on *Quercus* abundance in the eastern deciduous forest, USA: Are sapling densities most responsive to environmental gradients? Forest Ecology and Management, 201(2–3), 241–258. 10.1016/j.foreco.2004.06.023

[ece35216-bib-0007] Condit, R. , Ashton, P. S. , Baker, P. , Bunyavejchewin, P. , Gunatilleke, S. , Gunatilleke, S. , … Yamakura, T. (2000). Spatial patterns in the distribution of tropical tree species. Science, 288, 1414–1418. 10.1126/science.288.5470.1414 10827950

[ece35216-bib-0008] Dang, C. L. , Wang, C. Y. , & Wang, B. R. (2002). Succession and stability in plant community. Chinese Journal of Ecology, 21(2), 30–35 (in Chinese).

[ece35216-bib-0009] Fan, W. , Guo, H. , Wang, X. , & Duan, R. (2014). The effects of microhabitat, plant litter, and seed burial on the regeneration of *Quercus wutaishanica* and *Pinus tabulaeformis* . Scandinavian Journal of Forest Research, 29(2), 183–192. 10.1080/02827581.2014.885563

[ece35216-bib-0010] Feroz, S. M. , Yoshimura, K. , & Hagihara, A. (2008). Stand stratification and woody species diversity of a subtropical forest in limestone habitat in the northern part of Okinawa Island. Journal of Plant Research, 121, 329–337. 10.1007/s10265-008-0162-z 18425691

[ece35216-bib-0011] Fibich, P. , Leps, J. , Novotny, V. , Klimeš, P. , & Těšitel, J. (2016). Spatial patterns of tree species distribution in New Guinea primary and secondary lowland rain forest. Journal of Vegetation Science, 27(2), 328–339. 10.1111/jvs.12363

[ece35216-bib-0012] Gao, X. , & Sun, S. (2005). Effects of the small forest carnivores on the recruitment and survival of Liaodong oak (*Quercus wutaishanica*) seedlings. Forest Ecology and Management, 206(1), 283–292. 10.1016/j.foreco.2004.11.007

[ece35216-bib-0013] Getis, A. , & Franklin, J. (1987). Second‐order neighborhood analysis of mapped point pattern. Ecology, 68(3), 473–477. 10.2307/1938452

[ece35216-bib-0014] Getzin, S. , Dean, C. , He, F. , Trofymow, J. A. , Wiegand, K. , & Ecography, T. W. (2006). Spatial patterns and competition of tree species in a chronosequence of Douglas‐fir forest on Vancouver Island. Ecography, 29(5), 671–682. 10.1111/j.2006.0906-7590.04675

[ece35216-bib-0015] Gomez, J. M. (2004). Importance of microhabitat and acorn burial on *Quercus ilex* early recruitment: Non‐additive effects on multiple demographic processes. Plant Ecology, 172(2), 287–297. 10.1023/B:VEGE.0000026327.60991.f9

[ece35216-bib-0016] Greig‐Smith, P. (1952). Ecological observations on degraded and secondary forest in Trinidad, BritishWest Indies: II. Structure of the communities. Journal of Ecology, 40(2), 316–330. 10.2307/2256802

[ece35216-bib-0017] Guo, R. , Li, F. , He, W. , Yang, S. , & Sun, G. (2011). Spatial and temporal variability of annual precipitation during 1958–2007 in Loess Plateau, China. In D. Li, Y. Liu, & Y. Chen (Eds.), Computer and computing technologies in agriculture IV (pp. 551–560). Springer.

[ece35216-bib-0018] Harms, K. E. , Condit, R. , Hubbell, S. P. , & Foster, R. B. (2001). Habitat associations of trees and shrubs in a 50 ha neotropical forest plot. Journal of Ecology, 89(6), 947–959. 10.1111/j.1365-2745.2001.00615.x

[ece35216-bib-0019] Halpin, C. R. , & Lorimer, C. G. (2016). Trajectories and resilience of stand structure in response to variable disturbance severities in northern hardwoods. Forest Ecology and Management, 365, 69–82. 10.1016/j.foreco.2016.01.016

[ece35216-bib-0020] He, F. , Legendre, P. , & LaFrankie, J. V. (1997). Distribution patterns of tree species in a Malaysia tropical forest. Journal of Vegetation Science, 8(1), 105–114. 10.2307/3237248

[ece35216-bib-0021] Hubbell, S. P. , Ahumada, J. A. , & Condit, R. (2001). Local neighbourhood effects on long‐term survival of individual trees in a neotropical forest. Ecological Research, 5, 859–875. 10.1046/j.1440-1703.2001.00445.x

[ece35216-bib-0022] Jang, W. , Christopher, R. K. , & Lim, J. H. (2013). Application of mathematical models in the spatial analysis of early tree seedling distribution patterns within a tree fall gap at Gwangneung experimental forest. Journal of Plant Biology, 56(5), 283–289. 10.1007/s12374-013-0044-3

[ece35216-bib-0023] Janík, D. , Král, K. , Adam, D. , Horta, L. , Pavel, S. , Unara, P. , Vrskaa, T. , & McMahonb, S. (2016). Tree spatial patterns of Fagus sylvatica expansion over 37 years. Forest Ecology and Management, 375, 134–145. 10.1016/j.foreco.2016.05.017

[ece35216-bib-0024] Koukoulas, S. , & Blackburn, G. A. (2005). Spatial relationships between tree species and gap characteristics in broad‐leaved deciduous woodland. Journal of Vegetation Science, 16(5), 587–596. 10.1111/j.1654-1103.2005.tb02400.x

[ece35216-bib-0025] Larson, A. J. , Lutz, J. A. , Donato, D. C. , Freund, J. A. , Swanson, M. E. , Lambers, J. H. , … Franklin, J. F. (2016). Spatial aspects of tree mortality strongly differ between young and old‐growth forests. Ecology, 96(11), 2855–2861. 10.1890/15-0628.1 27070005

[ece35216-bib-0026] Lei, L. P. , Wang, X. A. , Guo, H. , & Zhu, Z. H. (2007). Dominant species of regeneration niche in *Quercus liaotungensis* and *Pinus tabulaeformis* forest in Ziwuling Mountain. Acta Botanica Boreal ‐ Occidental Sinica, 27(7), 1446–1453.

[ece35216-bib-0027] Leibold, M. A. (1995). The niche concept revisited: Mechanistic models and community context. Ecology, 76, 1371–1382. Chinese. 10.2307/1938141

[ece35216-bib-0028] Levin, S. A. (1992). The problem of pattern and scale in ecology. Ecology, 73(6), 1943–1967. 10.2307/1941447

[ece35216-bib-0029] Li, D. Z. , Shi, Q. , Zang, R. G. , Wang, X. Q. , Sheng, L. J. , Zhu, Z. L. , & Wang, C. (2006). Models for niche breadth and niche overlap of species or populations. Scientia Silvae Sincae, 42(7), 95–103.

[ece35216-bib-0030] Li, Y. D. , Xu, H. , Chen, D. X. , Luo, T Sh , Mo, J. H. , & Luo, W. (2008). Division of ecological species groups and functional groups based on interspecific association—A case study of the tree layer in the tropical lowland rainforest of Jianfengling in Hainan Island, China. Frontiers of Forestry in China, 3(4), 407–415.

[ece35216-bib-0031] López, P. P. , Alcázar, D. L. , & Ruiz, F. Z. (2010). Spatial pattern analysis of dominant species in the Prepuna: Gaining insight into community dynamics in the semi‐arid, subtropical Andes. Journal of Arid Environments, 74, 1534–1539. 10.1016/j.jaridenv.2010.06.008

[ece35216-bib-0032] Magurran, A. E. (1988). Ecological diversity and its measurement (p. 179). Princeton, NJ: Princeton University Press.

[ece35216-bib-0033] Martíneza, I. , Wieganda, T. , Taboadab, F. G. , Fernando, G. , & Joséramón, O. (2010). Spatial associations among tree species in a temperate forest community in North‐western Spain. Forest Ecology and Management, 260(4), 456–465. 10.1016/j.foreco.2010.04.039

[ece35216-bib-0034] Moeur, M. (1993). Characterizing spatial patterns of trees using stem‐mapped data. Forest Science, 39(4), 756–775.

[ece35216-bib-0035] Myster, R. W. , & Pickett, S. T. A. (1992). Effect of palatability and dispersal mode on spatial patterns of tree seedlings in old field. Bulletin of the Torrey Botanical Club, 119, 125–132.

[ece35216-bib-0036] Nakashizuka, T. (2001). Species coexistence in temperate, mixed deciduous forests. Trends in Ecology & Evolution, 16(4), 205. 10.1016/S0169-5347(01)02117-6 11245944

[ece35216-bib-0037] Noma, N. (1997). Annual fluctuations of sap fruits production within and inter species in a warm temperate forest on Yakushima Island. Tropics, 6, 441–449.

[ece35216-bib-0038] O'Hara, K. L. (2014). Multiaged silviculture: Managing for complex forest stand structures (p. 213). Oxford, UK: Oxford University Press.

[ece35216-bib-0039] Oliver, C. D. , & Larson, B. C. (1996). Forest stand dynamics. New York, NY: John Wiley & Sons.

[ece35216-bib-0040] Pacala, S. W. (1997). Dynamics of plant communities. In M. C. Crawley (Ed.), Plant ecology (2nd ed., pp. 532–555). Oxford, UK: Blackwell Scientific.

[ece35216-bib-0041] Pedersen, R. O. , Bollandsas, O. M. , Gobakken, T. , & Naesset, E. (2012). Deriving individual tree competition indices from airborne laser scanning. Forest Ecology and Management, 280, 150–265. 10.1016/j.foreco.2012.05.043

[ece35216-bib-0042] Pickett, S. T. A. (1989). Space‐for‐time substitution as an alternative to long‐term studies. In G. E. Likens (Ed.), Long‐term studies in ecology (pp. 110–135). New York, NY: Springer.

[ece35216-bib-0043] Rentch, J. S. , Fajvan, M. A. , & Rrjr, H. (2003). Spatial and temporal disturbance characteristics of oak‐dominated old‐growth stands in the central hardwood forest region. Forest Science, 49(5), 778–789.

[ece35216-bib-0044] Ripley, B. D. (1977). Modeling spatial pattern. Journal of the Royal Statistical Society, 39(2), 172–212.

[ece35216-bib-0045] Salas, C. , Lemay, V. , Nunez, P. , Pacheco, P. , & Espinosa, A. (2006). Spatial patterns in an old‐growth Nothofagus obliqua forest in south‐central Chile. Forest Ecology and Management, 231(1), 38–46. 10.1016/j.foreco.2006.04.037

[ece35216-bib-0046] Schleicher, J. , Wiegand, K. , & Ward, D. (2011). Changes of woody plant interaction and spatial distribution between rocky and sandy soil areas in a semi‐arid savanna, South Africa. Journal of Arid Environments, 75(3), 270–278. 10.1016/j.jaridenv.2010.10.003

[ece35216-bib-0047] Schurr, F. M. , Bossdorf, O. , Milton, S. , & Schumacher, J. (2004). Spatial pattern information in semi‐arid shrubland: A priori predicted versus observed pattern characteristics. Plant Ecology, 173(2), 271–282. 10.1023/B:VEGE.0000029335.13948.87

[ece35216-bib-0048] Shi, X. Z. , Yu, D. S. , Sun, W. X. , Wang, H. J. , Zhao, Q. G. , & Gong, Z. T. (2004). Reference benchmarks relating to great groups of genetic soil classification of China with soil taxonomy. Chinese Science Bulletin, 49(14), 1507–1511. 10.1360/03wd0476

[ece35216-bib-0049] Shibata, M. , Kikuchi, S. , Tanaka, H. , Sueyoshi, M. , & Yoshimaru, H. (2009). Effects of population density, sex morph, and tree size on reproduction in a heterodichogamous maple *Acer mono*, in a temperate forest of Japan. Ecological Research, 24(1), 6410–9. 10.1007/s11284-008-0474-4

[ece35216-bib-0050] Stoyan, D. , & Penttinen, A. (2000). Recent applications of point process methods in forestry statistics. Statistical Science, 15(1), 61–78. 10.1126/science.1193771

[ece35216-bib-0051] Su, S. J. , Liu, J. F. , He, Z. S. , Zheng, S. Q. , Hong, W. , & Xu, D. W. (2015). Ecological species group and interspecific association of dominant tree species in Daiyun Mountain Natural Reserve. Journal of Mountain Science, 12(3), 637–646. 10.1007/s11629-013-2935-7

[ece35216-bib-0052] Sun, W. Y. , Song, X. Y. , Mu, X. M. , Gao, P. , Wang, F. , & Zhao, G. J. (2015). Spatiotemporal vegetation cover variations associated with climate change and ecological restoration in Loess Plateau. Agricultural and Forest Meteorology, 210, 87–99. 10.1016/j.agrformet.2015.05.002

[ece35216-bib-0053] Tanner, J. E. , Hughes, T. P. , & Connell, J. H. (1994). Species coexistence, keystone species, and succession: A sensitivity analysis. Ecology, 8, 2204–2219. 10.2307/1940877

[ece35216-bib-0054] Tilman, D. (1994). Competition and biodiversity in spatially structured habitats. Ecology, 75(1), 2–16.

[ece35216-bib-0055] Tiphaine, D. , Hugo, A. , Frédérik, D. , & Yves, B. (2014). Structural and spatial characteristics of old‐growth temperate deciduous forests at their northern distribution limit. Forest Science, 60(5), 871–880. 10.5849/forsci.13-105

[ece35216-bib-0056] Thioulouse, J. , Chessel, D. , Doledec, S. , & Olivier, J. M. (1997). ADE‐4: A multivariate analysis and graphical display software. Statistics and Computing, 7(1), 75–83. 10.1023/a:1018513530268

[ece35216-bib-0057] Wiegand, T. , & Moloney, K. A. (2004). Rings, circles, and null‐models for point pattern analysis in ecology. Ookos, 104, 209–229. 10.1016/S0378-4274(02)00008-5

[ece35216-bib-0058] Wang, Y. F. (1991). Vegetation resources and their rational utilization on the Loess Plateau. Beijing, China: China Science and Technology Press.

[ece35216-bib-0059] Wang, D. L. , Li, W. Z. , & Cao, Z. (2016). Spatial pattern o*f Quercus wutaishanica* in natural secondary forest of Honglong Mountains. Acta Ecologica Sinica, 36(9), 6410–11 (in Chinese).

[ece35216-bib-0060] Wang, K. B. , Shao, R. X. , & Shangguan, Z. P. (2010). Changes in species richness and community productivity during succession on Loess Plateau in China. Polish Journal of Ecology, 58(3), 549–558.

[ece35216-bib-0061] Whitfeld, T. J. S. , Lasky, J. R. , Damas, K. , Sosanika, G. , Molem, K. , & Montgomery, R. A. (2014). Species richness, forest structure and functional diversity during succession in the New Guinea lowlands. Biotropica, 46(5), 538–548. 10.1111/btp.12136

[ece35216-bib-0062] Whitford, W. G. (2003). Ecology of desert systems. Journal of Mammalogy, 3, 1122–1124. 10.1644/1545-1542

[ece35216-bib-0063] Wiegand, K. , Jeltsch, F. , & Ward, D. (2000). Do spatial effects play a role in the spatial distribution of desert‐dwelling Acacia raddiana? Journal of Vegetation Science, 11(4), 473–484. 10.2307/3246577

[ece35216-bib-0064] Wiegand, T. , Moloney, K. A. , Naves, J. , & Knauer, F. (1999). Finding the missing link between landscape structure and population dynamics: A spatially explicit perspective. American Naturalist, 154(6), 605–627. 10.1086/303272 10600609

[ece35216-bib-0065] Xu, D. M. , Xu, X. Z. , & Xie, Y. Z. (2012). Dynamics of sandy desertification and detection of sandy land/steppe boundary: Vegetation and soil properties. Polish Journal of Ecology, 60(2), 251–263.

[ece35216-bib-0066] Yang, J. , Vázquez, L. , Feng, L. , Liu, Z. , & Zhao, G. (2018). Climatic and soil factors shape the demographical history and genetic diversity of a deciduous oak (*Quercus liaotungensis*) in Northern China. Frontiers in Plant Science, 9, 1534. 10.3389/fpls.2018.01534 30410498 PMC6209687

[ece35216-bib-0067] Yoda, K. , Kira, T. , Ogawa, H. , & Hozumi, K. (1963). Intraspecific competition among plants XI self‐thinning in overcrowded pure stands under cultivated and natural conditions. Journal of Biology, Osaka City University, 14, 107–129.

[ece35216-bib-0068] Yousef, E. , & Krzysztof, S. (2017). Intra‐ and interspecific interactions of Scots pine and European beech in mixed secondary forests. Acta Oecologica, 78, 15–25. 10.1016/j.actao.2016.12.002

[ece35216-bib-0069] Zhao, H. Y. , Kang, X. G. , Guo, Z. Q. , Yang, H. , & Xu, M. (2012). Species interactions in spruce‐fir mixed stands and implications for enrichment planting in the Changbai Mountains, China. Mountain Research and Development, 2, 187–196. 10.1659/MRD-JOURNAL-D-11-00125.1

[ece35216-bib-0070] Zhao, Z. Q. , Wang, L. H. , Bai, Z. K. , Pan, Z. , & Wang, Y. (2015). Development of population structure and spatial distribution patterns of a restored forest during 17‐year succession (1993–2010) in Pingshuo opencast mine spoil, China. Environmental Monitoring and Assessment, 7, 6410–13. 10.1007/s10661-015-4391-z 26071680

[ece35216-bib-0071] Zhang, L. J. , Gove, J. H. , Liu, C. M. , & Leak, W. B. (2001). A finite mixture of two Weibull distributions for modeling the diameter distributions of rotated sigmoid, uneven‐aged stands. Journal of Forest Research, 31, 1654–1659. 10.1139/cjfr-31-9-1654

[ece35216-bib-0072] Zhu, Z Ch (1999). The type and succession of the *Quereus laiotungensis* forests on woodland of Loess Plateau in north Shaanxi Province. Journal of Northwest Forestry College, 21(1), 57–71.

[ece35216-bib-0073] Zou, H. Y. , Cheng, J. M. , & Lei, L. (1998). Natural recoverage succession and regulation of the prairie vegetation on the Loess Plateau. Research of Soil and Water Conservation, 5(1), 126–138.

[ece35216-bib-0074] Zou, H. Y. , Liu, G. B. , & Wang, H. S. , (2002). The vegetation development in North Ziwulin forest region in last fifty years. Acta Botanica Boreal ‐ Occidental Sinica, 22(1), 6410–8.

[ece35216-bib-0075] Zou, T. , Li, Y. , Xu, H. , & Xu, G. Q. (2010). Responses to precipitation treatment for Haloxylon ammodendron growing on contrasting textured soils. Ecological Research, 25(1), 185–194. 10.1007/s11284-009-0642-1

